# Circ_0032821 acts as an oncogene in cell proliferation, metastasis and autophagy in human gastric cancer cells in vitro and in vivo through activating MEK1/ERK1/2 signaling pathway

**DOI:** 10.1186/s12935-020-1151-0

**Published:** 2020-03-06

**Authors:** Yuanyuan Jiang, Yan Zhang, Feifei Chu, Lidong Xu, Huili Wu

**Affiliations:** Department of Gastroenterology, Zhengzhou University Affiliated Zhengzhou Central Hospital, No. 195, Tongbai Road, Zhengzhou, 450007 Henan China

**Keywords:** Circ_0032821, Proliferation, Metastasis, Autophagy, Gastric cancer

## Abstract

**Background:**

Circular RNA (circRNA) is increasingly attracting attention in gastric cancer (GC). Hsa_circ_0032821 (circ_0032821) has been declared to be upregulated in human GC tissues. However, the biological role of circ_0032821 remains undisclosed in GC cells.

**Methods:**

Expression of circ_0032821 was measured by real-time quantitative PCR. Cell proliferation, autophagy, Epithelial-mesenchymal transition (EMT), migration, and invasion were evaluated by Cell counting kit-8 assay, western blotting or transwell assays. Expression of proliferating cell nuclear antigen (PCNA), Matrix metalloproteinase 2 (MMP2), MMP9, Light chain 3 (LC3), p62, total and phosphorylated Extracellular signal-regulated kinase 1/2 (ERK1/2) and Mitogen-activated protein kinase’s kinase 1 (MEK1) was evaluated by western blotting. Xenograft tumor model was established to measure tumor growth in vivo.

**Results:**

Circ_0032821 was significantly upregulated in human GC tumors and cells. Moreover, circ_0032821 might be a biomarker for the advanced Tumor node metastasis (TNM) stage, lymphoid node metastasis and poor prognosis in gastric cancer. Knockdown of circ_0032821 by transfection induced decrease of cell proliferation, EMT, migration and invasion, but increase of autophagy of AGS and HGC-27 cells in vitro, as well as induced tumor growth inhibition in vivo. Besides, overexpression of circ_0032821 by transfection functioned the opposite effects in human GC cells. Mechanically, the MEK1/ERK1/2 signaling pathway was activated when circ_0032821 upregulation, whereas inhibited when circ_0032821 silencing.

**Conclusion:**

Circ_0032821 expression induced cell proliferation, EMT, migration, invasion, and autophagy inhibition in human GC cells in vitro and in vivo through activating MEK1/ERK1/2 signaling pathway, suggesting circ_0032821 as an oncogenic role in GC.

## Highlights

1. Circ_0032821 was upregulated in human gastric cancer tumors and cells.
2. Circ_0032821 was associated with advanced TNM stage, lymphoid node metastasis and poor survival in gastric cancer.
3. Circ_0032821 contributed to the malignancy of gastric cancer cells in vitro and in vivo by promoting cell proliferation, EMT, migration and invasion, and inhibiting autophagy.
4. MEK1/ERK1/2 signaling pathway was activated by circ_0032821 in gastric cancer cells.

## Background

Gastric cancer (GC) is the third tumor mortality worldwide [1]. The prognosis of GC patients is inversely proportional to the cancer stage. Even though over 90% patients with early GC survive for 5 years, the advanced GC patients harbor a poor prognosis statistically [2]. Moreover, the incidence of GC remains high in China, whereas less than 20% GC patients are diagnosed at early stages [3, 4]. In considering the treatment, the only radical therapy for GC remains surgery nowadays [5]. However, tumor invasiveness and distant metastasis always happen after operation, thus limiting its efficiency [6]. Therefore, it is essential to explore the deep mechanisms responsible for GC metastasis, and to investigate new targets for the detection of early GC.

Circular RNAs (circRNAs) are newly discovered endogenous, circular transcripts from genetic loci [7]. It has been annotated that circRNAs are another class of noncoding RNAs (ncRNAs), and are widespread in varying tissues, cells and circulating systems including plasma. It is characterized by covalently closed continuous loops [8, 9], without 5′ caps or 3′ poly (A) tails which exist in liner message RNAs (mRNAs). Over the past 3 years, dysregulation of circRNA profiles have been published, as well as the role in GC progression [10, 11]. Very recently, circRNAs have been documented as biomarkers and targets for human cancers including GC [12, 13]. Since the stability of circRNAs is higher than linear splicing products such as miRNAs, long noncoding RNAs and mRNAs, circRNAs could be a promising potential biomarkers [10]. However, biological roles of circRNAs in cancers including GC remain to be fully uncovered.

According to Gene expression omnibus database (GSO, GSE78092), hundreds of circRNAs including hsa_circ_0032821 (circ_0032821) were deregulated in human GC tissues than non-GC tissues. Whereas, the detail cellular behaviors of circ_0032821 is unclear. Here, we detected expression of circ_0032821 in human GC tissue samples, and its role in cell proliferation, migration, invasion, Epithelial-mesenchymal transition (EMT) and autophagy in human GC cells in vitro, as well as tumor growth in vivo. Mechanically, we assessed the activation of Mitogen-activated protein kinase’s kinase (MEK)/Extracellular signal-regulated kinase (ERK) signaling pathway.

## Materials and methods

### Clinical tissue samples

The study included a total of 60 patients with GC from Zhengzhou University Affiliated Zhengzhou Central Hospital. These patients were received none chemoradiotherapy before this surgery. All manipulates involved in this study were approved by the Ethics Committee of Zhengzhou University Affiliated Zhengzhou Central Hospital in paper, and agreed by every patient in the form of written consent. All the GC tissues were collected from surgical specimens, as well as the paired adjacent normal tissues, which were localized at 5 cm away from the edge of the GC sites. All the GC tissues and normal tissues were further confirmed by pathological analysis, then stored in liquid nitrogen.

### Cell culture

One normal human gastric epithelial mucosa cell line (GES-1) was obtained from the Cancer Institute and Hospital of the Chinese Academy of Medical Sciences (Beijing, China). Five GC cell lines AGS (CRL-1739) and SNU-1 (CRL-5971) were primary purchased from the American Type Culture Collection (Manassas, VA, USA); HGC-27(94042256) was from European Collection of Authenticated Cell Cultures (Public Health England; Porton Down, Salisbury, UK); MKN74 (JCRB0255) and MKN1 (JCRB0252) were from Japanese Collection of Research Bioresources Cell Bank (Ibaraki city, Osaka, Japan). All of these cells were cultured in Dulbecco’s modified eagle medium (DMEM; Gibco, Gaithersburg, MD, USA) medium plus 10% Fetal bovine serum (FBS; Gibco) and 1% penicillin/streptomycin in a humidified atmosphere of 5% CO_2_ at 37 °C. AGS and HGC-27 cells were exposed to Ly294002 (10 μM, Sigma-Aldrich) or Rapamycin (100 nM) for 1 day.

### Real-time quantitative polymerase chain reaction (RT-qPCR)

Total RNA in tissue samples and cultured cells was extracted by TRIzol (Life Technologies, Carlsbad, CA, USA) following the manufacturer’s protocol. The RNA was firstly transferred into complementary DNA (cDNA) using Bestar™ qPCR RT kit (DBI Bioscience, Ludwigshafen, Germany), and cDNA was then amplified using Bestar™ qPCR MasterMix (DBI Bioscience). The gene expression was assessed using special paired primers on CFX 96 Touch system (Bio-Rad, Hercules, CA, USA). Glyceraldehyde-3-phosphate dehydrogenase (GAPDH) was used as internal control of circ_0032821. The primers of circ_0032821 and GAPDH were listed as follows: circ_0032821, 5′-AGGAATCTGAGTTGCAGTGTCTC-3′ (forward) and 5′-TGATCCTTGAGCTGCAATCTGG-3′ (reversed) and GAPDH, 5′-GGAGCGAGATCCCTCCAAAAT-3′ (forward) and 5′-GGCTGTTGTCATACTTCTCATGG-3′ (reversed). All experiments were performed in triplicate. The value of threshold cycle (Ct) was recorded to analyze the relative gene expression on RNA level using 2^−ΔΔCt^ method.

### Cell transfection

Small interfering RNA (siRNA) against circ_0032821 (si-circ_0032821) and its negative control siRNA (si-NC) were from GenePharma (Shanghai, China). AGS and HGC-27 cells were seeded in 6-well plates for overnight. For overexpression, pcDNA vectors containing circ_0032821 (circ_0032821 vector) was constructed. Then, 100 nM of siRNA or 2 μg of vectors were transfected into cells using Lipofectamine RNAiMax (Life Technologies) obeyed the instructions. After days, the transfected cells were collected for further analysis.

### Cell Counting Kit (CCK)-8 assay

Transfected AGS and HGC-27 cells (4 × 10^3^ cells/well) were re-seeded in 96-well plates, and cell proliferation was evaluated by CCK-8 assay. After incubation for 0 day, 1 day, 2 days and 3 days, 10 μL of CCK-8 solution (Dojindo, Kumamoto, Japan) was added into each well for another 2 h. The absorbance at 450 nm was measured using a microplate reader (Bio-Rad). The cell proliferative curve was drawn.

### Transwell migration and invasion assays

To evaluate abilities of cell migration and invasion, transfected AGS and HGC-27 cells were cultured in non-coated or matrigel-coated transwell chambers (8 μm, BD Biosciences, Franklin Lakes, NJ, USA), respectively. Briefly, after transfection for 1 day, the 3 × 10^3^ cells were re-suspended in 200 μL FBS-free DMEM medium; whereas, the lower chamber was filled with 400 μL DMEM supplemented with 20% FBS. Next, the chambers were incubated at 37 °C and 5% CO_2_ for 1 day, followed by 0.5% crystal violet staining. The migrated and invaded cells were imaged under an inverted microscope (200×), and five random fields were captured.

### Western blotting

Tissue samples and cultured cells were lysed with RIPA buffer (Life Technologies) to harvest total cellular proteins. Markers of proliferation, invasion, EMT, autophagy, and MEK1/ERK1/2 signaling pathway were examined using western blotting. The western blotting produces were performed as described previously [14]. The special primary antibodies included Proliferating cell nuclear antigen (PCNA; ab152112, 1:2000), Matrix metalloproteinase 2 (MMP2; ab2462, 1:1000), MMP9 (ab76003; 1:10000), E-cadherin (ab15148, 1:500), N-cadherin (ab98952; 1:1000), Vimentin (ab137321, 1:2000), Light chain 3 (LC3; ab 51520, 1:2000), p62 (ab155686, 1:2000), total MEK1 (t-MEK1; ab139343, 1:2000), phosphorylated MEK1 (p-MEK1; ab254096, 1:1000), total ERK1/2 (t-ERK1; ab36991, 1:5000), phosphorylated ERK1/2 (p-ERK1/2; ab214362, 1:500), and β-actin (ab49900; 1:50000). The relative protein expression level was analyzed on Image J with β-actin as endogenous control.

### Animal experiments

AGS cells were stably transfected with shRNA target circ_003281/NC (sh-circ_003281/NC) or vectors containing circ_0032821 or not. The shRNAs were purchased from GenePharma. Afterwards, the transfected AGS cells (4 × 10^6^ cells) in the logarithmic growth phase were subcutaneously injected into the right flank of female BALB/c nude mice (n = 4, 4–6-week-old). The mice were purchased from Beijing Vital River Laboratory Animal Technology Co., Ltd. (Beijing, China), and all operations were following the Guideline of Care and Use of Laboratory Animals. This in vivo experiment was approved by the Animal Care and Use Committee of Zhengzhou University Affiliated Zhengzhou Central Hospital. After injection, the length and width of xenograft tumors were measured every week, and the tumor volume was calculated using the formula: 0.5 × length × width^2^. Five weeks later, the mice were euthanized by dislocation of cervical vertebrae. The tumors were dissected and weighted. Then, tumors were stored in − 80 °C for further total RNA and protein extraction.

### Statistical analysis

Two-tailed Student’s *t* test was used to calculate statistical significance between two groups. The overall survival time was analyzed by Kaplan–Meier analysis. All data were presented as mean ± standard deviation and analyzed using the SPSS 16.0 (SPSS, Chicago, IL, USA). Data with *P *< 0.05 were considered statistically significant.

## Results

### Expression of circ_0032821 was upregulated in human GC tissue and cells

**Fig. 1:**
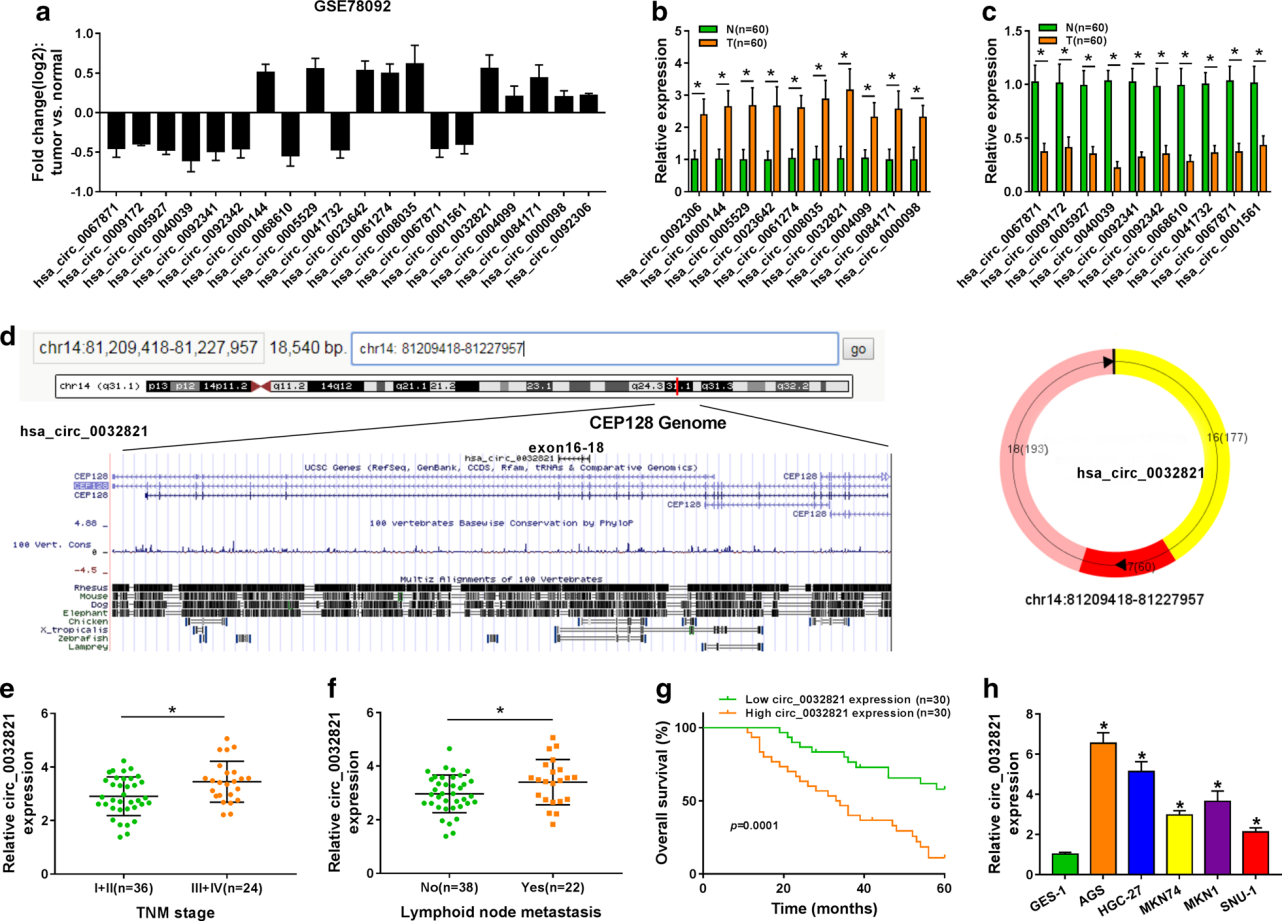
Expression of hsa_circ_003281 (circ_0032821) was upregulated in human gastric cancer (GC) tissue and cells. **a** Ten top upregulated circRNAs and ten downregulated circRNAs were presented according to Gene Expression Omnibus database (GEO, GSE78092). **b**, **c** These 20 circRNAs were detected in this cohort of gastric cancer patients (n = 60) using RT-qPCR. N, normal adjacent tissue; T, tumor tissue. **d** The schematic diagram of genomic location of circ_0032821. **e** RT-qPCR detected circ_0032821 levels in GC tumor tissues at low Tumor node metastasis (TNM) stage (I + II, n = 36) and advanced TNM stage (III + IV, n = 24). **f** RT-qPCR detected circ_0032821 levels in primary GC tumor tissues (n = 38) and metastatic tumor tissues in lymphoid node (n = 22). **g** Kaplan–Meier survival plots analyzed the overall survival rate of this cohort of GC patients with high (≥ Median, n = 30) or low (< Median, n = 30) expression of circ_0032821. **h** Circ_0032821 expression level was tested in the human GC cell lines (AGS, HGC-27, MKN74, MKN1, and SNU-1) and normal gastric epithelial cells GES-1. *P < 0.05

Firstly, we analyzed the published RNA-seq data of human GC tissues and matched normal gastric tissues. According to GSE78092 dataset, the top ten upregulated and ten downregulated circRNAs were presented as shown in Fig. 1a. Afterwards, these 20 circRNAs were further identified in a cohort of GC patients (n = 60) using RT-qPCR. The data showed that these putative circRNAs were significantly upregulated or downregulated in line with GSE78092 dataset (Fig. 1b, c). Meanwhile, expression of circ_003281 was the highest among these 10 upregulated circRNAs in these cases. Therefore, we selected circ_003281 to investigate its role in human GC. The circBase (http://circrna.org/) depicted that circ_003281 was derived from exons 16, 17 and 18 of CEP128 gene (Fig. 1d). Besides, we observed even higher level of circ_003281 in advanced GC tumors (n = 24) and metastatic lymphoid nodes (n = 22) (Fig. 1e, f). Kaplan–Meier analysis demonstrated that patients with high expression of circ_003281 (≥ median) were remarkably associated with poor overall survival rate (Fig. 1g). Besides, expression of circ_003281 was overall higher in five human GC cell lines AGS, HGC-27, MKN74, MKN1 and SNU-1 than that in normal gastric epithelial cell line GES-1 (Fig. 1h). These results indicated that circ_003281 was upregulated in human GC tissues and cells, and this upregulation might be associated with poor prognosis of GC patients.

### Knockdown of circ_0032821 suppressed cell proliferation, EMT, migration and invasion in human GC cells in vitro

**Fig. 2:**
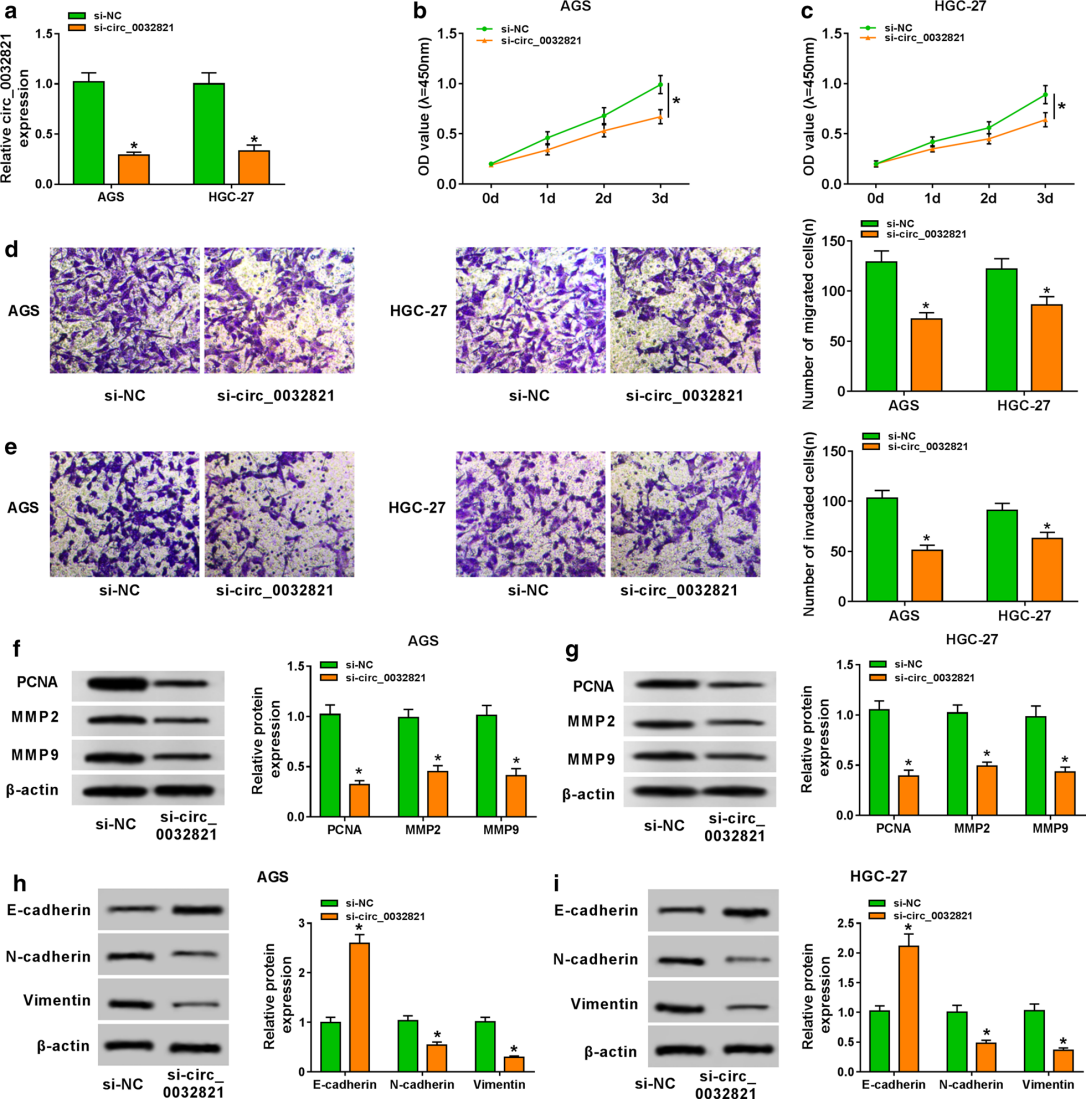
Knockdown of circ_0032821 suppressed cell proliferation, epithelial-mesenchymal transition (EMT), migration and invasion in human GC cells in vitro. AGS and HGC-27 cells were transfected with siRNA against circ_0032821 (si-circ_0032821) or its scrambled sequence (si-NC). **a** RT-qPCR detected circ_0032821 level after transfection for 1 day. **b**, **c** Cell proliferative ability was assessed by Cell counting kit (CCK)-8 after transfection for 3 days. Optimal density (OD) values at 450 nm were recorded at 0 day, 1 day, 2 days, and 3 days. **d**, **e** Cell migratory and invasive abilities were evaluated by transwell assays. The migrated cells and invaded cells were counted after transfection for 1 day. One representative image (×200) in each group was presented. **f**, **g** Western blotting measured protein expression of proliferating cell nuclear antigen (PCNA), matrix metalloproteinase 2 (MMP2) and MMP9 in transfected AGS and HGC-27 cells on 1 day. **h**, **i** Western blotting measured protein expression of E-cadherin, N-cadherin and Vimentin in transfected AGS and HGC-27 cells on 1 day. Relative protein expression was normalized to β-actin. *P < 0.05

In order to explore the potential biological role of circ_0032821 in GC cells, we transiently transfected si-circ_0032821 or si-NC into AGS and HGC-27 cells. Then, the silencing efficiency was measured by RT-qPCR, and circ_0032821 level was dramatically decreased in si-circ_0032821-transfected cells (Fig. 2a). Subsequently, a series of functional analyses were carried out. CCK-8 assay assessed that cell proliferative ability of AGS and HGC-27 cells was reduced after si-circ_0032821 transfection for 3 day (Fig. 2b, c). Transwell assays showed that knockdown of circ_0032821 attenuated cell migratory and invasive capacities of AGS and HGC-27 cells after 1 day transfection (Fig. 2d, e). Moreover, declined PCNA, MMP2 and MMP9 (markers of proliferation and invasion) were induced in the presence of si-circ_0032821 for 1 day (Fig. 2f, g). In terms of EMT, E-cadherin was greatly induced, whereas N-cadherin and Vimentin were distinctively inhibited in AGS and HGC-27 cells which were knocked down circ_0032821 expression for 1 day (Fig. 2h, i). These findings demonstrated a suppressive effect of circ_0032821 knockdown on cell proliferation, EMT, migration and invasion of human GC cells in vitro.

### Knockdown of circ_0032821 promoted cell autophagy of human GC cells in vitro

**Fig. 3:**
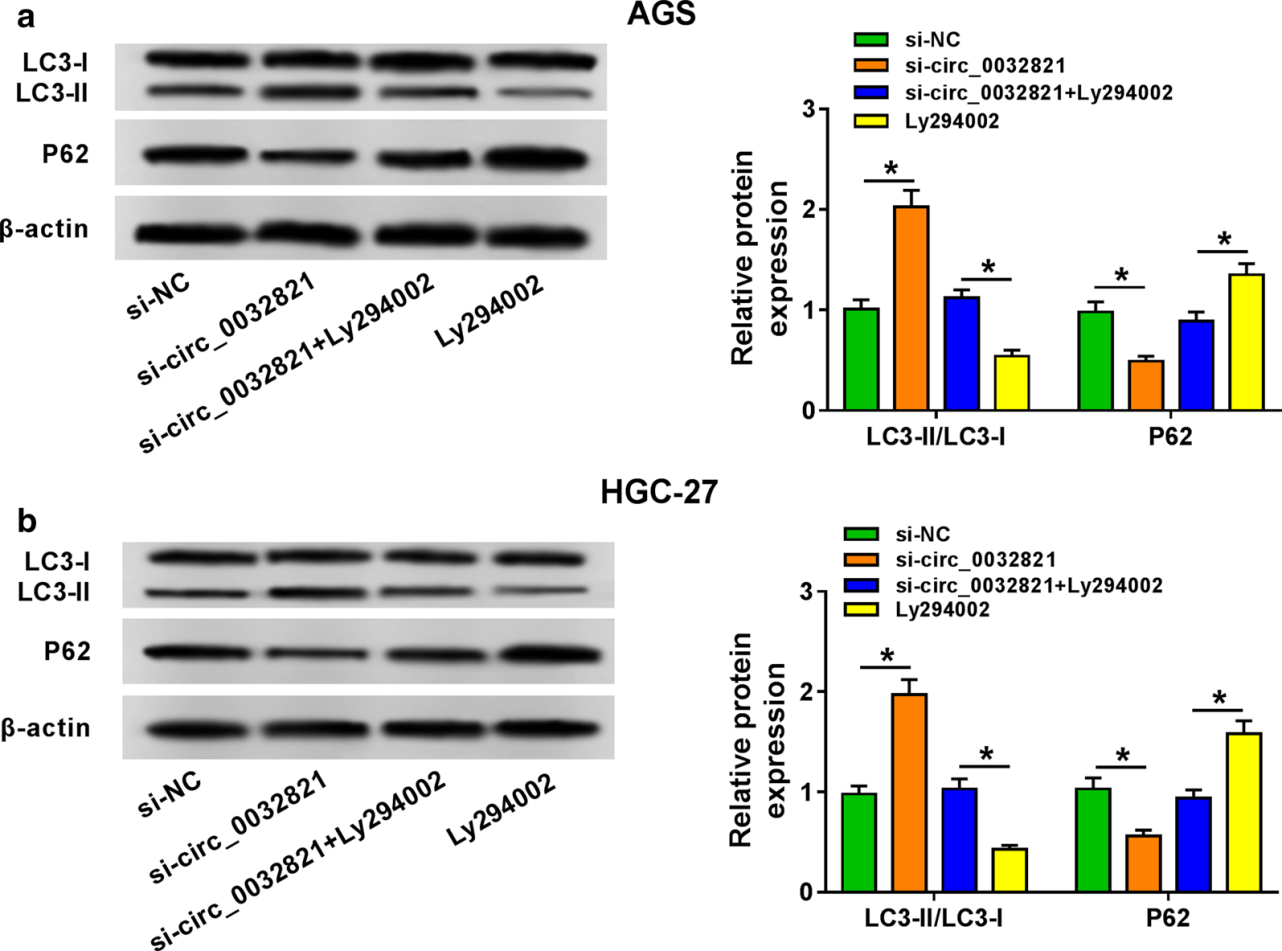
Knockdown of circ_0032821 promoted cell autophagy of human GC cells in vitro. AGS and HGC-27 cells were transfected with si-circ_0032821, followed with treatment of Ly294002 (autophagy inhibitor, 10 μM) for 1 day or not. **a**, **b** Western blot detection of autophagy-related proteins LC3-I, LC3-II and p62. *P < 0.05

In quantifying autophagy, western blotting analyses were performed to examine expression status of autophagy-related proteins LC3 and p62. With transfection, si-circ_0032821 caused an elevation of LC3-II/LC3-I ratio, and a decrease of p62 level in AGS and HGC-27 cells than its control si-NC (Fig. 3a, b); the influence of circ_0032821 knockdown on LC3 and p62 expression was abated with treatment of autophagy inhibitor Ly294002 (10 μM, 1 day). This result supported the tumor-suppressive effect of circ_0032821 knockdown on autophagy of human GC cells in vitro. Taken Figs. 2 and 3 together, circ_0032821 knockdown could exert anti-tumor role in human GC cells in vitro.

### Overexpression of circ_0032821 facilitated cell proliferation, EMT, migration and invasion in human GC cells in vitro

**Fig. 4:**
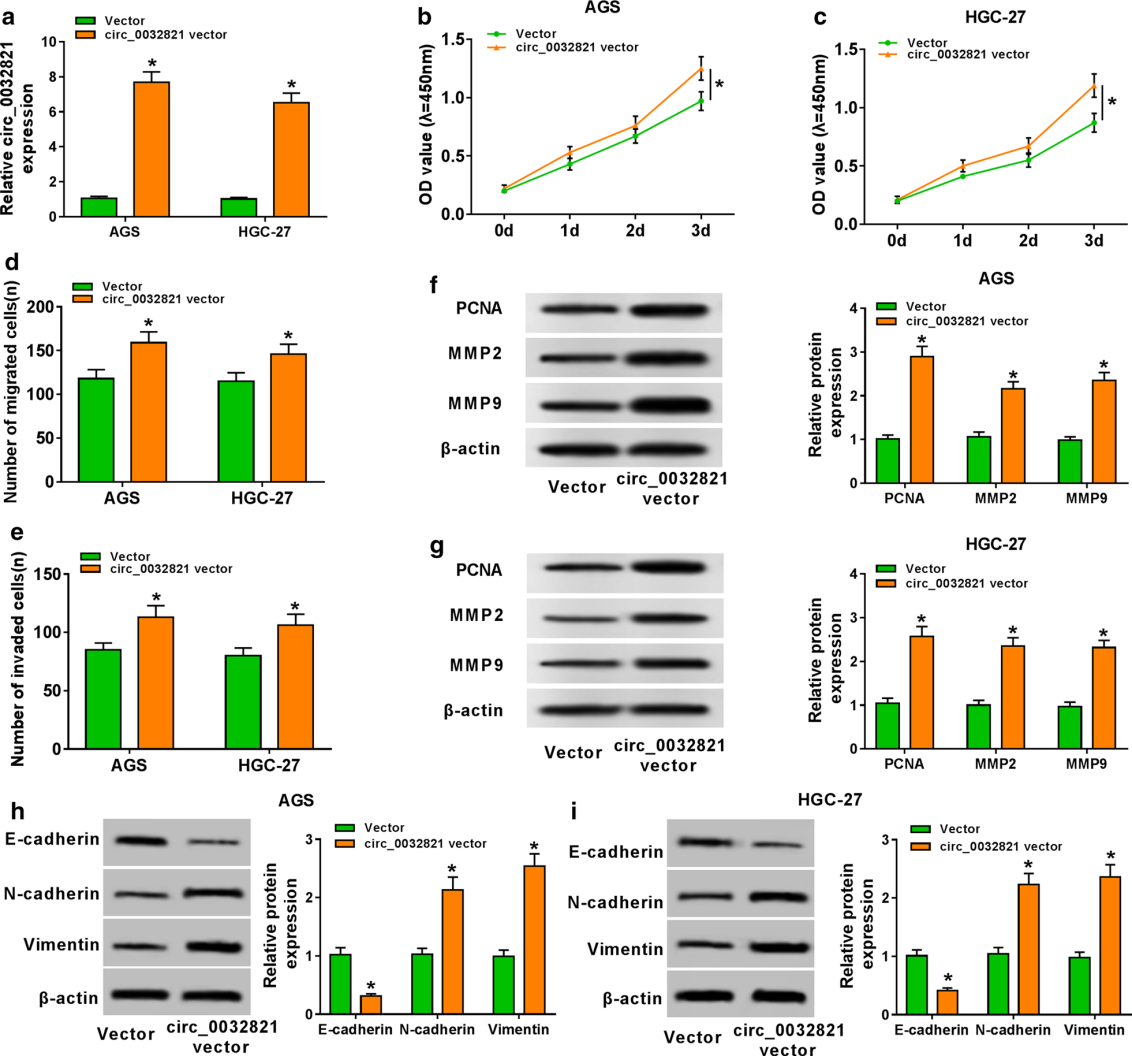
Overexpression of circ_0032821 facilitated cell proliferation, EMT, migration and invasion in human GC cells in vitro. AGS and HGC-27 cells were transfected with circ_0032821 overexpression vector (circ_0032821 vector) or the empty vectors (Vector). **a** RT-qPCR detected circ_0032821 level after transfection for 1 day. **b**, **c** Cell proliferative ability was assessed by CCK-8 after transfection for 3 days. **d**, **e** Cell migratory and invasive abilities were evaluated by transwell assays. Western blotting measured protein expression of **f**, **g** PCNA, MMP2 and MMP9, and **h**, **i** E-cadherin, N-cadherin and Vimentin in transfected AGS and HGC-27 cells on 1 day. *P < 0.05

To explore the potential biological role of circ_0032821 in GC cells, we also did gain-of-function experiments in AGS and HGC-27 cells transfected with circ_0032821 overexpressing vectors or empty vectors. After transfection for 1 day, RT-qPCR analysis showed that circ_0032821 was abundantly higher expressed in AGS and HGC-27 cells, which suggested a high overexpression efficiency (Fig. 4a). CCK-8 assay assessed that cell proliferative ability of AGS and HGC-27 cells was promoted after circ_0032821 vector transfection for 3 days (Fig. 4b, c), accompanied with raised PCNA expression (Fig. 4f, g). Transwell assays showed that overexpression of circ_0032821 enhanced cell migratory and invasive capacities of AGS and HGC-27 cells after 1 day transfection (Fig. 4d, e), accompanied with raised MMP2 and MMP9 expression (Fig. 4f, g). In addition, EMT was also facilitated when circ_0032821 was forcedly upregulated, as described by dropped E-cadherin level, and elevated N-cadherin and Vimentin levels (Fig. 4h, i). These findings demonstrated a promoting effect of circ_0032821 overexpression on cell proliferation, EMT, migration and invasion of human GC cells in vitro.

### Overexpression of circ_0032821 inhibited cell autophagy of human GC cells in vitro

**Fig. 5:**
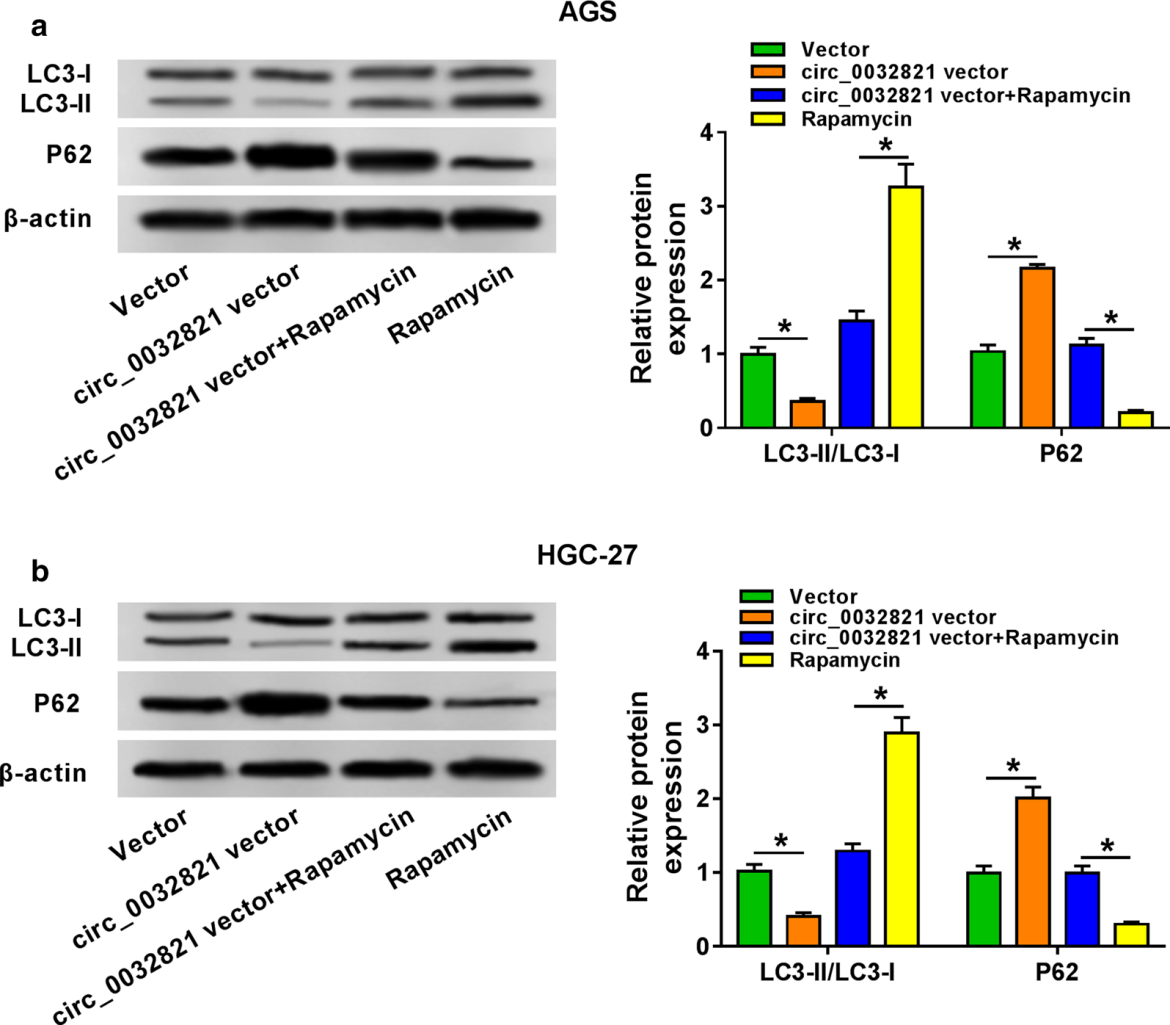
Overexpression of circ_0032821 inhibited cell autophagy of human GC cells in vitro. AGS and HGC-27 cells were transfected with circ_0032821 vector, followed with treatment of Rapamycin (autophagy promotor) for 1 day or not. **a**, **b** Western blot detection of LC3-I, LC3-II and p62. *P < 0.05

Furthermore, the ratio of LC3-II/LC3-I was diminished, and p62 expression was augmented in circ_0032821-upregulated AGS and HGC-27 cells (Fig. 5a, b), which were counteracted by treatment of autophagy enhancer Rapamycin (100 nM, 1 day). This result supported the suppressive effect of circ_0032821 overexpression on autophagy of human GC cells in vitro. Taken Figs. 4 and 5 together, circ_0032821 overexpression could function oncogenic role in human GC cells in vitro.

### Circ_0032821 positively modulated MEK1/ERK1/2 signaling pathway in human GC cells

**Fig. 6:**
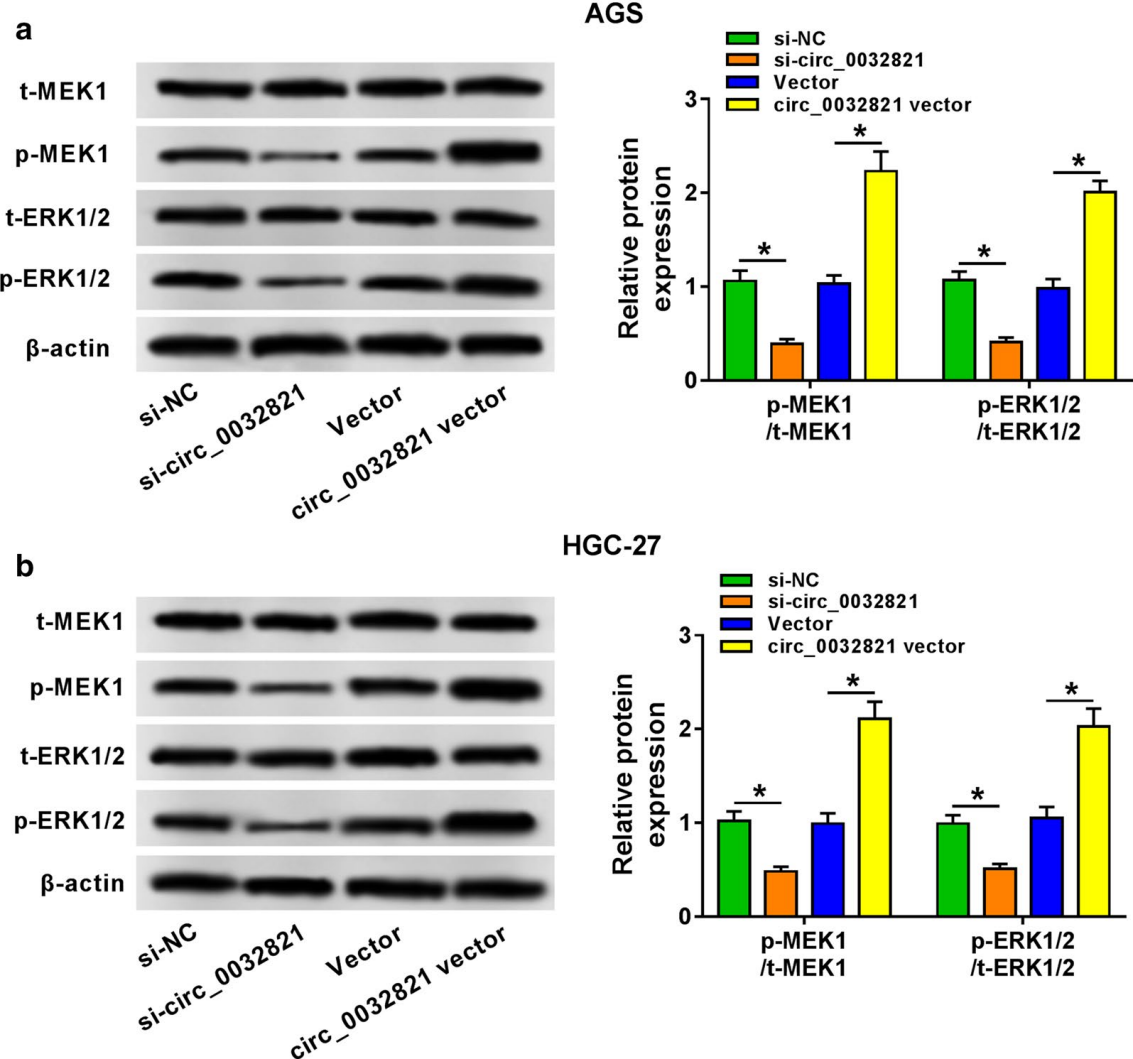
Circ_0032821 positively modulated MEK1/ERK1/2 signaling pathway in human GC cells. AGS and HGC-27 cells were transfected with si-circ_0032821, circ_0032821 vector and their negative controls. **a**, **b** Western blot detected total MEK1 and ERK1/2 (t-MEK1 and t-ERK1/2) and phosphorylated MEK1 and ERK1/2 (p-MEK1 and p-ERK1/2) after transfection for 1 day. *P < 0.05

Through the foregoing data, circ_0032821 served as an oncogene in GC. The MEK1/ERK1/2 signaling pathway was further researched using western blotting. Relative expression of p-MEK1 and p-ERK1/2 was depressed by si-circ_0032821 transfection, and facilitated by circ_0032821 vector transfection in AGS and HGC-27 cells (Fig. 6a, b). This outcome indicated circ_0032821 expression could contribute to the activation of MEK1/ERK1/2 signaling pathway.

### Circ_0032821 functioned as an oncogene in cell growth and MEK1/ERK1/2 signaling pathway in human GC cells in vivo

**Fig. 7:**
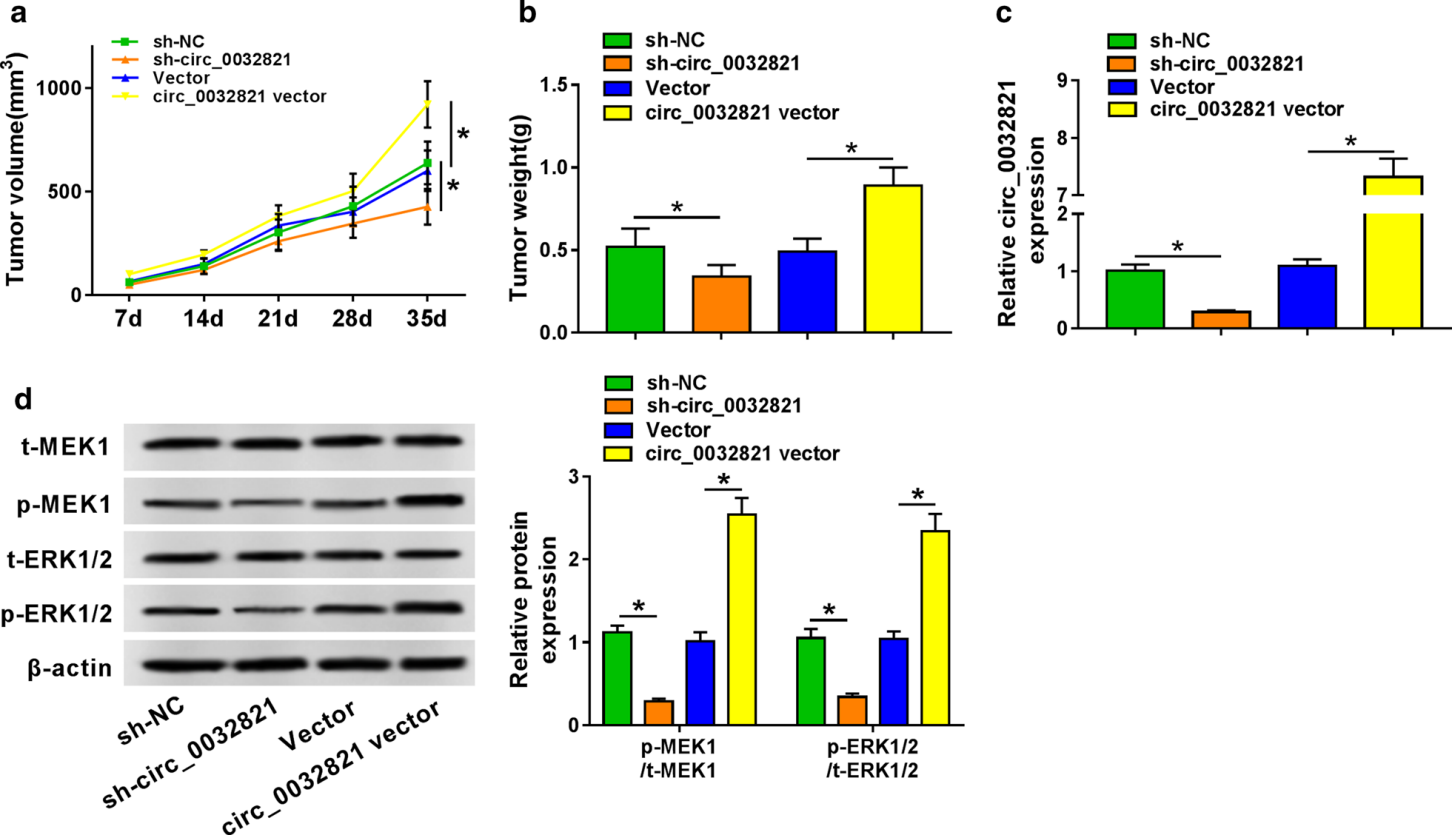
Circ_0032821 functioned as an oncogene in tumor growth and MEK1/ERK1/2 signaling pathway in human GC cells in vivo. AGS cells were stably transfected with sh-circ_0032821, circ_0032821 vector and their negative controls. **a** Tumor volume was calculated every 7 days after implantation of transfected AGS cells into nude mice (n = 4). **b** Tumor weight was measured after implantation for 35 days. **c** RT-qPCR detected circ_0032821 expression in xenograft tumor tissues (n = 4). **d** Western blot examined t-MEK1, t-ERK1/2, p-MEK1 and p-ERK1/2 levels in xenograft tumor tissues (n = 4). One representative western blot image in each group was presented. *P < 0.05

The xenograft mice models were conducted to verify whether circ_0032821 functioned an oncogene in GC cells in vivo. With construction of stably transfected sh-circ_0032821 or circ_0032821 vector, AGS cells were subcutaneously injected into nude mice (n = 4). As depicted in Fig. 7a, tumor volumes and tumor growth curve were hindered in sh-circ_0032821-transfected mice, but promoted in circ_0032821 vector-transfected mice. Meanwhile, after xenograft tumor dissection, tumor weight was reduced when circ_0032821 was knocked down, and was increased when circ_0032821 was overexpressed (Fig. 7b, c). Meanwhile, consistently with the in vitro results, circ_0032821 expression could positively modulate MEK1/ERK1/2 signaling pathway in AGS-induced GC tumors in vivo (Fig. 7d). These results suggested that circ_0032821 contributed to GC tumorigenesis partially through activating MEK1/ERK1/2 signaling pathway.

## Discussion

GC is still a serious threat in recent society due to its distant metastasis and deficiency of early biomarker for diagnosis. Emerging evidences have been depicted that circRNAs, another class of ncRNA, are rising stars in cancers including GC [15]. Moreover, the rapid development of circRNA research becomes a blowout phenomenon in recent 3 years. Dozens of circRNAs have been announced to participate in the progression of GC cells. For example, the novel circ_0000144, circOSBPL10 and circHECTD1 were upregulated in GC, and silencing of them could inhibit human GC cell proliferation, migration and invasion in vitro and in vivo [16–18]. The other tumor-related physiological processes had also been modulated by dysregulation of circRNAs, such as cell apoptosis, autophagy, Warburg effect, glutaminolysis, EMT, and cisplatin resistance, as well as the underlying signaling pathways [18–21]. Moreover, several circRNAs were suggested as new noninvasive biomarkers in early GC, such as circ_0006848, circ_0065149 and circ-KIAA1244 [22–24]. Inspired by these, we aimed to search and verify a novel circRNA that could contribute to GC carcinogenesis.

We firstly analyzed the published GEO database and GSE78092 dataset. The top ten differently expressed circRNAs were selected to test their expression level in this cohort of GC patients. According to RT-qPCR data, relative expression of circ_0032821 (about 3.12 fold, Fig. 1b) was the highest one and circ_0040039 (about 0.25 fold, Fig. 1c) was the lowest one in GC tissues comparing to the adjacent normal tissues. However, Huang et al. [25] noticed that circ_0008035 was higher expressed than circ_0032821, which was inconsistent with our finding. Expression of circ_0040039 was also observed largely downregulated in GC tissues from their patient cases. These differences might happen because of the different tissue samples. In that research, they claimed the tumor-promoting role of circ_0008035 in GC cell proliferation and invasion in vitro via miR-375/Y-box binding protein-1 axis. Here, we illustrated an oncogenic activity of circ_0032821 in GC cells on cell proliferation, EMT, autophagy, migration and invasion through MEK1/ERK1/2 signaling pathway, as well as on tumor growth in vivo. Furthermore, high expression of circ_0032821 in GC tissues was associated with advanced TNM stage, lymphoid node metastasis and shorter survival time.

Mechanically, accumulating data considered that circRNAs can regulate gene expression in cancers through sponging microRNAs (miRNAs) and RNA-binding proteins, and functioning as translation templates [10]. Besides, the circRNA-miRNA-mRNA network was proposed to be involved in histological classification and disease progression in GC [26]. Whereas, this present study was defective about the exploration of the molecular mechanism hidden circ_0032821 through serving as a molecular sponge, and this would be further solved. Besides, we detected circ_0032821 expression in GC tissues and cells, without in plasmas. In order to make circ_0032821 as a noninvasive/circulating biomarker for GC detection, we’d better to further monitor serum circ_0032821 in GC patients versus normal control volunteers in the near further. Moreover, it is imperative to discuss correlations of early clinical manifestations and circ_0032821 expression in serum or tissue.

Except ceRNA regulatory network, signaling pathway network was another essential mechanism of circRNAs. AKT1/mTOR, β-catenin/c-myc, and hippo signaling pathways had been claimed to be complicated in the role of circRNA in GC cells [18, 20, 27]. Here, we analyzed the activation of RAS-RAF-MEK-ERK MAPK signaling pathway, a well-documented pathway in human cancers [28]. Expression of p-MEK1 and p-ERK1/2 was inhibited by circ_0032821 silencing, and facilitated by circ_0032821 overexpression in human GC cells both in vitro and in vivo. Similarly, another circRNA circDLST was also discovered to promote GC tumorigenesis and metastasis by activating NRAS/MEK1/ERK1/2 pathway [29]. Circ_0032821 was identified to be upregulated in GC tissues and cells, which suggested an increased activation of MEK1/ERK1/2 axis. And this finding was in line with the notion that ERK1/2 activation was pivotal to tumor progression [30]. In addition, acting as downstream targets of MAPKs, MMP2 and MMP9 were also highly induced when circ_0032821 was upregulated in GC cells, thus causing the cancer cell invasion.

## Conclusion

Collectively, we demonstrated that circ_0032821 was upregulated in human GC tissues, and that circ_0032821 functioned an oncogenic role in GC cell proliferation, EMT, autophagy, migration and invasion in vitro, and tumor growth in vivo by activating MEK1/ERK1/2 axis. This work may provide a scientific evidence about circ_0032821 as a potential biomarker and target for the diagnosis and prognosis of GC patients.

## Data Availability

All data generated or analyzed during this study are included in this published article.
